# Supplementary material for: A comprehensive investigation of intracortical and corticothalamic models of the alpha rhythm
Source: PLoS Comput Biol. 2025 Apr 10;21(4):e1012926. doi: 10.1371/journal.pcbi.1012926 (PMC12064047; doi:10.1371/journal.pcbi.1012926)
Supplement: S1 Appendix — Details the derivation process leading to the formulation of the second-order differential equation used to represent the PSP output. (PDF) [file pcbi.1012926.s001.pdf]

S1 Appendix. Derivation of the JR Model Equations

The Jansen-Rit and related models are often discussed in terms of a convolution integral for the synaptic impulse response function, as well as the corresponding equivalent second-order differential equation, which is typically what is used in numerical simulations. The mathematical relationship between these is however rarely given in literature sources, and so we provide that here, with a full derivation of the JR differential equation from its impulse response, using the Laplace transform as it simplifies convolution operations by turning them into algebraic manipulations in the Laplace domain.

The synaptic impulse response is defined as an alpha function, which is described by the following equations:

$$h(t) = \begin{cases} \alpha\beta t e^{-\beta t}, & t \geq 0 \\ 0 & \text{otherwise} \end{cases} \tag{1}$$

with α as the maximum amplitude of the PSP and β the rate constant parameter. The first step consists of finding the Laplace transform of $h(t)$, denoted as $H(s)$, which is defined as follows:

$$\begin{aligned}
H(s) = \mathcal{L}\{h(t)\} &= \int_0^\infty h(t)e^{-st}\,dt && (2)\\
&= \int_0^\infty \alpha\beta t e^{-\beta t} e^{-st}\,dt && (3)\\
&= \int_0^\infty \alpha\beta t e^{(-\beta-s)t}\,dt && (4)\\
&= \lim_{b\to\infty}\left[\int_0^b \alpha\beta t e^{(-\beta-s)t}\,dt\right] && (5)\\
&= \lim_{b\to\infty}\left(\left[\alpha\beta t\frac{1}{-\beta-s}e^{(-\beta-s)t}\right]_0^b - \int_0^b \frac{\alpha\beta}{-\beta-s}e^{(-\beta-s)t}\,dt\right) && (6)\\
&= \frac{\alpha\beta}{-\beta-s}\lim_{b\to\infty}\left(be^{(-\beta-s)b} - \int_0^b e^{(-\beta-s)t}\,dt\right) && (7)\\
&= \frac{\alpha\beta}{\beta+s}\lim_{b\to\infty}\int_0^b e^{(-\beta-s)t}\,dt && (8)\\
&= \frac{\alpha\beta}{\beta+s}\lim_{b\to\infty}\left[\frac{1}{-\beta-s}e^{(-\beta-s)t}\right]_0^b && (9)\\
&= \frac{\alpha\beta}{(\beta+s)^2}\lim_{b\to\infty}\left[1-e^{(-\beta-s)b}\right] && (10)\\
&= \frac{\alpha\beta}{(\beta+s)^2} && (11)
\end{aligned}$$

Now, with an expression for $H(s)$ in the Laplace domain, and given that $y(t)$ is equal to

the convolution of $h(t)$ and $x(t)$, we can represent this relationship in the Laplace domain as:

$$\begin{aligned} Y(s) &= X(s)H(s) && (12) \\ Y(s) &= X(s)\frac{\alpha\beta}{(\beta+s)^2} && (13) \\ (\beta+s)^2 Y(s) &= X(s)\alpha\beta && (14) \\ s^2Y(s) + \beta^2 Y(s) + 2\beta s Y(s) &= \alpha\beta X(s) && (15) \\ s^2 Y(s) &= \alpha\beta X(s) - 2\beta s Y(s) - \beta^2 Y(s) && (16) \end{aligned}$$

Since $s^2Y(s)$ corresponds to the second derivative in the time domain, translating equation (40) back into the time domain, we obtain:

$$\ddot{y}(t) = \alpha\beta x(t) - 2\beta\dot{y}(t) - \beta^2 y(t) \quad (17)$$

This corresponds to the commonly used JR second-order differential equation, which can be rewritten in the form of two first-order ODE's:

$$\begin{aligned} \dot{y}(t) &= z(t) && (18) \\ \dot{z}(t) &= \alpha\beta x(t) - 2\beta z(t) - \beta^2 y(t) && (19) \end{aligned}$$

with $y(t)$ representing the average postsynaptic membrane potential (output of the PSP block).
