# Supplementary material for: A comprehensive investigation of intracortical and corticothalamic models of the alpha rhythm
Source: PLoS Comput Biol. 2025 Apr 10;21(4):e1012926. doi: 10.1371/journal.pcbi.1012926 (PMC12064047; doi:10.1371/journal.pcbi.1012926)
Supplement: S2 Appendix — Presents the transfer function equations for each model along with their corresponding outputs. (PDF) [file pcbi.1012926.s002.pdf]

S2 Appendix. Transfer Function of the Models

In addition to comparing the numerical simulation outputs, we also simulated the analytical (or linearized) power spectra of the models. These analytical models are used for stability analysis, offering clear insights into how a system responds to changes in inputs. Moreover, they provide explicit solutions, enhancing our understanding of the system's behavior. The transfer functions for both the JR and MDF models are derived from graph control analysis. The equations for the LW model are sourced from Hartoyo et al. (2019) [1], while those for the RRW model are from Robinson et al. (2002) [2].

JR Transfer Function:

$$T(s) = \frac{G_e}{1 + K_s^2 G_e (C_3 C_4 G_i - C_1 C_2 G_e)}$$

with:

$$G_e = \frac{Aa^{-1}}{(a^{-1} + s)^2},\ G_i = \frac{Bb^{-1}}{(a^{-1} + s)^2},\ \text{and } K_s = \frac{e_0 r}{2}$$

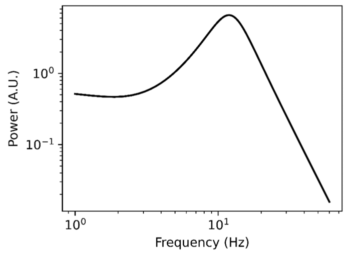

MDF Transfer Function:

$$T(s) = \frac{G_e^2 K_s \gamma_2 \,(1 + K_s G_i \gamma_5)}{1 + K_s G_i \gamma_5 + G_e G_i K_s^2 \gamma_3 \gamma_4 - G_e^2 K_s^2 \gamma_2 \gamma_1 \,(1 + K_s G_i \gamma_5)}$$

with:

$$G_e = \frac{H_e \kappa_e^{-1}}{(\kappa_e^{-1} + s)^2},\ G_i = \frac{H_i \kappa_i^{-1}}{\left(\kappa_i^{-1} + s\right)^2},\ \text{and } K_s = \frac{e_0 r}{2}$$

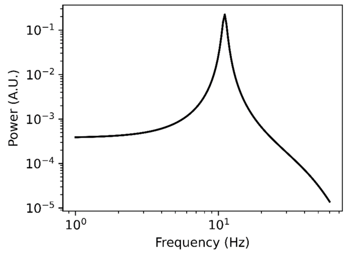

LW Transfer Function:

$$T(s) = \frac{H_1}{1 + H_1 H_2}$$

with:

$$H_1 = \frac{Q_{ee}}{Q_{ee} A_{ee} - K_{ei} Y_e},\ H_2 = \frac{Q_{ie} Q_{ei} A_{ei} A_{ie}}{Q_{ee}(K_{ie} Y_i - Q_{ii} A_{ii})},\ Q_{xy} = -\frac{\psi_{xy}(V_y)}{\tau_y},$$

$$A_{xy} = -\Gamma_x \gamma_x e N_{xy}^b S_x'(V_x), \qquad I_{xy} = \frac{\Gamma_x e}{\gamma_x} N_{xy}^b S_x(V_x) + \frac{\Gamma_x e}{\gamma_x} p_{xy},$$

$$Y_x = (s + \gamma_x)^2, \qquad K_{xy} = s + \frac{1}{\tau_x}\left(1 + \frac{I_{xx}}{|V_x^{eq} - V_x^{rest}|} + \frac{I_{yx}}{|V_y^{eq} - V_x^{rest}|}\right)$$

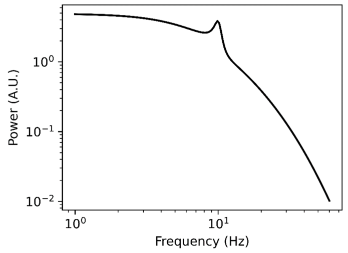

RRW Transfer Function:

$$T(S) = \frac{G_{es} G_{sn} L^2 e^{s t_0/2}}{(1 - G_{ei} L)(1 - G_{srs} L^2)(q^2 r_e^2)}$$

with:

$$L = \left(1 - \frac{s}{\alpha}\right)^{-1} \left(1 - \frac{s}{\beta}\right)^{-1}$$

$$q^2 r_e^2 = \left(1 - \frac{s}{\gamma_e}\right)^2 - \frac{L}{1 - G_{ei} L}\left(G_{ee} + \frac{G_{ese} L + G_{esre} L^2}{1 - G_{srs} L^2} e^{s t_0}\right)$$

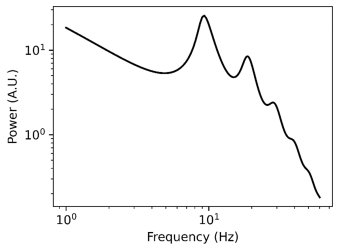

Fig A. ***Transfer function of each of the four studied models (JR, MDF, LW, and RRW) with the corresponding analytical power spectra*** The transfer function of JR and MDF were derived using graph control analysis. The derivations for LW transfer function are sourced from [1], and for RRW from [2]

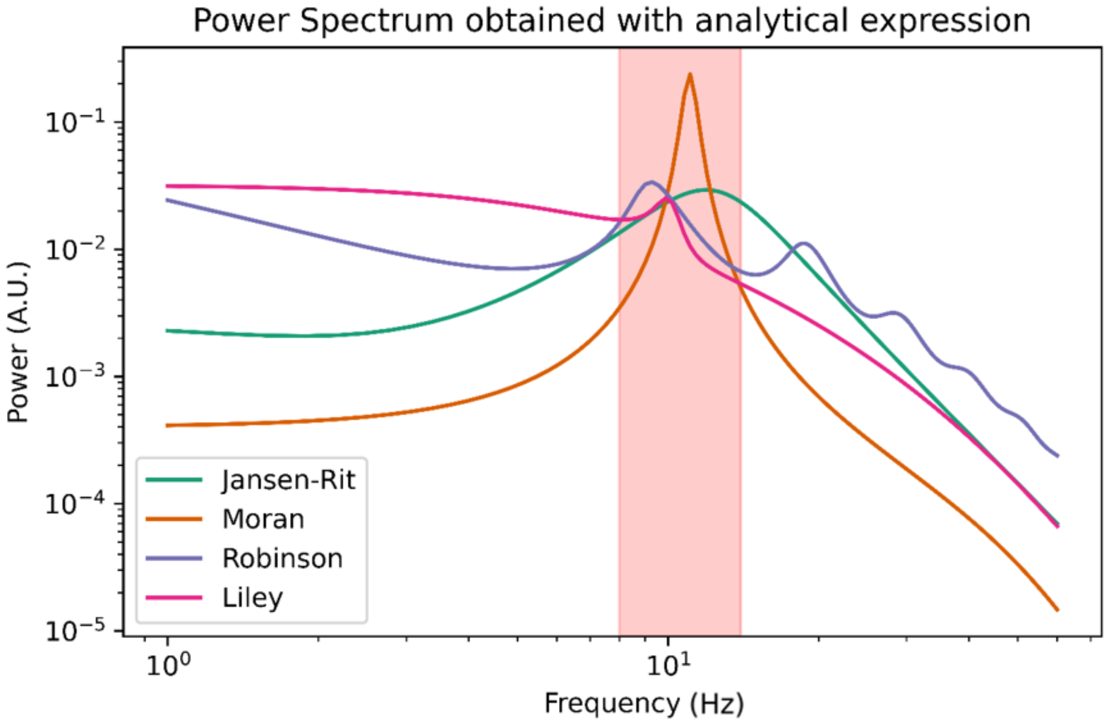

Fig B. ***Analytical power spectra of the four models together.*** After linearization, the models continue to generate alpha rhythms with a peak between 8-12 Hz (represented by the red zone). Among the models, RRW is unique in capturing the harmonics at higher frequencies in its linearized form. JR exhibits a broader peak compared to the others, while LW has a lower peak height.

References

[1] Hartoyo A, Cadusch PJ, Liley DT, Hicks DG. Parameter estimation and identifiability in a neural population model for electro-cortical activity. PLoS computational biology. 2019;15(5):e1006694.

[2] Robinson P, Rennie C, Rowe D. Dynamics of large-scale brain activity in normal arousal states and epileptic seizures. Physical Review E. 2002;65(4):041924.
