# Supplementary material for: A comprehensive investigation of intracortical and corticothalamic models of the alpha rhythm
Source: PLoS Comput Biol. 2025 Apr 10;21(4):e1012926. doi: 10.1371/journal.pcbi.1012926 (PMC12064047; doi:10.1371/journal.pcbi.1012926)
Supplement: S3 Appendix — Mathematical details of the derivation process to determine the fixed points of the system and study the stability. (PDF) [file pcbi.1012926.s003.pdf]

S3 Appendix. Derivation of Stability Analysis for JR and LW

For JR, similar to Grimbert et al. (2006) [1], the fixed points are determined by setting the derivatives to 0. With some manipulations, the equilibrium points in the $(C, y_1 - y_2)$ plane with $y = y_1 - y_2$ are equal to:

$$y = \frac{A}{a}p + \frac{A}{a}C_2 S(\frac{A}{a}C_1 S(y) - \frac{B}{b}C_4 S(\frac{A}{a}C_3 S(y)) \quad (1)$$

The stability of the fixed points is then defined using the Jacobian matrix

$$\mathbf{Y}_{i,j} = \begin{bmatrix} \frac{\partial y_0}{\partial y_0} & \frac{\partial y_0}{\partial y_1} & \frac{\partial y_0}{\partial y_2} & \frac{\partial y_0}{\partial y_3} & \frac{\partial y_0}{\partial y_4} & \frac{\partial y_0}{\partial y_5} \\ \frac{\partial y_1}{\partial y_0} & \frac{\partial y_1}{\partial y_1} & \frac{\partial y_1}{\partial y_2} & \frac{\partial y_1}{\partial y_3} & \frac{\partial y_1}{\partial y_4} & \frac{\partial y_1}{\partial y_5} \\ \frac{\partial y_2}{\partial y_0} & \frac{\partial y_2}{\partial y_1} & \frac{\partial y_2}{\partial y_2} & \frac{\partial y_2}{\partial y_3} & \frac{\partial y_2}{\partial y_4} & \frac{\partial y_2}{\partial y_5} \\ \frac{\partial y_3}{\partial y_0} & \frac{\partial y_3}{\partial y_1} & \frac{\partial y_3}{\partial y_2} & \frac{\partial y_3}{\partial y_3} & \frac{\partial y_3}{\partial y_4} & \frac{\partial y_3}{\partial y_5} \\ \frac{\partial y_4}{\partial y_0} & \frac{\partial y_4}{\partial y_1} & \frac{\partial y_4}{\partial y_2} & \frac{\partial y_4}{\partial y_3} & \frac{\partial y_4}{\partial y_4} & \frac{\partial y_4}{\partial y_5} \\ \frac{\partial y_5}{\partial y_0} & \frac{\partial y_5}{\partial y_1} & \frac{\partial y_5}{\partial y_2} & \frac{\partial y_5}{\partial y_3} & \frac{\partial y_5}{\partial y_4} & \frac{\partial y_5}{\partial y_5} \end{bmatrix} = \begin{bmatrix} 0 & 0 & 0 & 1 & 0 & 0 \\ 0 & 0 & 0 & 0 & 1 & 0 \\ 0 & 0 & 0 & 0 & 0 & 1 \\ -a^2 & AaS'(y) & -AaS'(y) & -2a & 0 & 0 \\ AaC_2C_1S'(C_1 y_0(y)) & -a^2 & 0 & 0 & -2a & 0 \\ BbC_4C_3S'(C_3 y_0(y)) & 0 & -b^2 & 0 & 0 & -2b \end{bmatrix}$$

with y corresponding to the fixed point of interest and $y_0(y) = \frac{A}{a}S(y)$. Stability is then defined by calculating the eigenvalues of the matrix $\mathbf{Y}$ for each fixed point, and looking at the sign of the real part of the eigenvalues. The system is stable if all the eigenvalues have a negative real part. If at least one of the eigenvalues has a positive real part, it is considered as an unstable fixed point.

Using a similar method (estimation of the fixed point, following an assessment of the stability of the fixed points by looking at the real part of the eigenvalues of the Jacobian matrix), the LW equilibrium points' stability was also determined. The full calculation and equations are detailed in the appendix of Hartoyo et al. (2019) [2] and also in S9 Appendix. Briefly:

The equilibrium point equations can be reduced to:

$$0 = -V_e + V_{er} + \psi_{ee}(V_e)I_{ee} + \psi_i e(V_e)I_{ie} \quad (2)$$

$$0 = -V_i + V_{ir} + \psi_{ei}(V_i)I_{ei} + \psi_{ii}(V_i)I_{ii} \quad (3)$$

with

$$I_{ee} = \frac{\Gamma_e e}{\gamma_e} N_{ee}^{\beta} S(V_e) + \frac{\Gamma_e e}{\gamma_e} p_{ee} \quad (4)$$

$$I_{ei} = \frac{\Gamma_e e}{\gamma_e} N_{ei}^{\beta} S(V_e) + \frac{\Gamma_e e}{\gamma_e} p_{ei} \quad (5)$$

$$I_{ie} = \frac{\Gamma_i e}{\gamma_i} N_{ie}^{\beta} S(V_i) + \frac{\Gamma_i e}{\gamma_i} \quad (6)$$

$$I_{ii} = \frac{\Gamma_i e}{\gamma_i} N_{ii}^{\beta} S(V_i) + \frac{\Gamma_i e}{\gamma_i} \quad (7)$$

The fixed points for V_e and V_i are then estimated by finding the values for which values these two equations intersect.

The Jacobian matrix is:

$$\mathbf{F}_{i,j} = \begin{bmatrix}
\frac{\partial V_e}{\partial V_e} & \frac{\partial V_e}{\partial V_i} & \frac{\partial V_e}{\partial I_{ee}} & \frac{\partial V_e}{\partial I_{ei}} & \frac{\partial V_e}{\partial I_{ie}} & \frac{\partial V_e}{\partial I_{ii}} & \frac{\partial V_e}{\partial U_{ee}} & \frac{\partial V_e}{\partial U_{ei}} & \frac{\partial V_e}{\partial U_{ie}} & \frac{\partial V_e}{\partial U_{ii}} \\
\frac{\partial V_i}{\partial V_e} & \frac{\partial V_i}{\partial V_i} & \frac{\partial V_i}{\partial I_{ee}} & \frac{\partial V_i}{\partial I_{ei}} & \frac{\partial V_i}{\partial I_{ie}} & \frac{\partial V_i}{\partial I_{ii}} & \frac{\partial V_i}{\partial U_{ee}} & \frac{\partial V_i}{\partial U_{ei}} & \frac{\partial V_i}{\partial U_{ie}} & \frac{\partial V_i}{\partial U_{ii}} \\
\frac{\partial I_{ee}}{\partial V_e} & \frac{\partial I_{ee}}{\partial V_i} & \frac{\partial I_{ee}}{\partial I_{ee}} & \frac{\partial I_{ee}}{\partial I_{ei}} & \frac{\partial I_{ee}}{\partial I_{ie}} & \frac{\partial I_{ee}}{\partial I_{ii}} & \frac{\partial I_{ee}}{\partial U_{ee}} & \frac{\partial I_{ee}}{\partial U_{ei}} & \frac{\partial I_{ee}}{\partial U_{ie}} & \frac{\partial I_{ee}}{\partial U_{ii}} \\
\frac{\partial I_{ei}}{\partial V_e} & \frac{\partial I_{ei}}{\partial V_i} & \frac{\partial I_{ei}}{\partial I_{ee}} & \frac{\partial I_{ei}}{\partial I_{ei}} & \frac{\partial I_{ei}}{\partial I_{ie}} & \frac{\partial I_{ei}}{\partial I_{ii}} & \frac{\partial I_{ei}}{\partial U_{ee}} & \frac{\partial I_{ei}}{\partial U_{ei}} & \frac{\partial I_{ei}}{\partial U_{ie}} & \frac{\partial I_{ei}}{\partial U_{ii}} \\
\frac{\partial I_{ie}}{\partial V_e} & \frac{\partial I_{ie}}{\partial V_i} & \frac{\partial I_{ie}}{\partial I_{ee}} & \frac{\partial I_{ie}}{\partial I_{ei}} & \frac{\partial I_{ie}}{\partial I_{ie}} & \frac{\partial I_{ie}}{\partial I_{ii}} & \frac{\partial I_{ie}}{\partial U_{ee}} & \frac{\partial I_{ie}}{\partial U_{ei}} & \frac{\partial I_{ie}}{\partial U_{ie}} & \frac{\partial I_{ie}}{\partial U_{ii}} \\
\frac{\partial I_{ii}}{\partial V_e} & \frac{\partial I_{ii}}{\partial V_i} & \frac{\partial I_{ii}}{\partial I_{ee}} & \frac{\partial I_{ii}}{\partial I_{ei}} & \frac{\partial I_{ii}}{\partial I_{ie}} & \frac{\partial I_{ii}}{\partial I_{ii}} & \frac{\partial I_{ii}}{\partial U_{ee}} & \frac{\partial I_{ii}}{\partial U_{ei}} & \frac{\partial I_{ii}}{\partial U_{ie}} & \frac{\partial I_{ii}}{\partial U_{ii}} \\
\frac{\partial U_{ee}}{\partial V_e} & \frac{\partial U_{ee}}{\partial V_i} & \frac{\partial U_{ee}}{\partial I_{ee}} & \frac{\partial U_{ee}}{\partial I_{ei}} & \frac{\partial U_{ee}}{\partial I_{ie}} & \frac{\partial U_{ee}}{\partial I_{ii}} & \frac{\partial U_{ee}}{\partial U_{ee}} & \frac{\partial U_{ee}}{\partial U_{ei}} & \frac{\partial U_{ee}}{\partial U_{ie}} & \frac{\partial U_{ee}}{\partial U_{ii}} \\
\frac{\partial U_{ei}}{\partial V_e} & \frac{\partial U_{ei}}{\partial V_i} & \frac{\partial U_{ei}}{\partial I_{ee}} & \frac{\partial U_{ei}}{\partial I_{ei}} & \frac{\partial U_{ei}}{\partial I_{ie}} & \frac{\partial U_{ei}}{\partial I_{ii}} & \frac{\partial U_{ei}}{\partial U_{ee}} & \frac{\partial U_{ei}}{\partial U_{ei}} & \frac{\partial U_{ei}}{\partial U_{ie}} & \frac{\partial U_{ei}}{\partial U_{ii}} \\
\frac{\partial U_{ie}}{\partial V_e} & \frac{\partial U_{ie}}{\partial V_i} & \frac{\partial U_{ie}}{\partial I_{ee}} & \frac{\partial U_{ie}}{\partial I_{ei}} & \frac{\partial U_{ie}}{\partial I_{ie}} & \frac{\partial U_{ie}}{\partial I_{ii}} & \frac{\partial U_{ie}}{\partial U_{ee}} & \frac{\partial U_{ie}}{\partial U_{ei}} & \frac{\partial U_{ie}}{\partial U_{ie}} & \frac{\partial U_{ie}}{\partial U_{ii}} \\
\frac{\partial U_{ii}}{\partial V_e} & \frac{\partial U_{ii}}{\partial V_i} & \frac{\partial U_{ii}}{\partial I_{ee}} & \frac{\partial U_{ii}}{\partial I_{ei}} & \frac{\partial U_{ii}}{\partial I_{ie}} & \frac{\partial U_{ii}}{\partial I_{ii}} & \frac{\partial U_{ii}}{\partial U_{ee}} & \frac{\partial U_{ii}}{\partial U_{ei}} & \frac{\partial U_{ii}}{\partial U_{ie}} & \frac{\partial U_{ii}}{\partial U_{ii}}
\end{bmatrix}$$

which evaluates to

$$\mathbf{F}_{i,j} = \begin{bmatrix}
G(V_e) & 0 & \frac{\psi_{ee}(V_e)}{\tau_e} & 0 & \frac{\psi_{ie}(V_e)}{\tau_e} & 0 & 0 & 0 & 0 & 0 \\
G_(V_i) & 0 & \frac{\psi_{ei}(V_i)}{\tau_i} & 0 & \frac{\psi_{ii}(V_i)}{\tau_i} & 0 & 0 & 0 & 0 & 0 \\
0 & 0 & 0 & 0 & 0 & 0 & 1 & 0 & 0 & 0 \\
0 & 0 & 0 & 0 & 0 & 0 & 0 & 1 & 0 & 0 \\
0 & 0 & 0 & 0 & 0 & 0 & 0 & 0 & 1 & 0 \\
0 & 0 & 0 & 0 & 0 & 0 & 0 & 0 & 0 & 1 \\
\Gamma_e\gamma_e e N_{ee}^{\beta} S'(V_e) & 0 & -\gamma_e^2 & 0 & 0 & 0 & -2\gamma_e & 0 & 0 & 0 \\
\Gamma_e\gamma_e e N_{ei}^{\beta} S'(V_e) & 0 & 0 & -\gamma_e^2 & 0 & 0 & 0 & -2\gamma_e & 0 & 0 \\
0 & \Gamma_i\gamma_i e N_{ie}^{\beta} S'(V_i) & 0 & 0 & -\gamma_i^2 & 0 & 0 & 0 & -2\gamma_i & 0 \\
0 & \Gamma_i\gamma_i e N_{ii}^{\beta} S'(V_i) & 0 & 0 & 0 & -\gamma_i^2 & 0 & 0 & 0 & -2\gamma_i
\end{bmatrix}$$

with

$$G(V_e) = \frac{1}{\tau_e}(-1 - \frac{I_{ee}}{|V_e^e q - V_{er}|} - \frac{I_{ie}}{|V_i^e q - V_{ir}|}) \quad (8)$$

$$G(V_i) = \frac{1}{\tau_i}(-1 - \frac{I_{ei}}{|V_e^e q - V_{ir}|} - \frac{I_{ii}}{|V_i^e q - V_{ir}|}) \quad (9)$$

We then replace V_e and V_i with the equilibrium points computed previously, and the real parts of the eigenvalues of this Jacobian matrix are then examined to assess their stability.

References

[1] Grimbert F, Faugeras O. Bifurcation analysis of Jansen's neural mass model. Neural computation. 2006;18(12):3052–3068

[2] Hartoyo A, Cadusch PJ, Liley DT, Hicks DG. Parameter estimation and identifiability in a neural population model for electro-cortical activity. PLoS computational biology. 2019;15(5):e1006694.
