# Supplementary material for: A comprehensive investigation of intracortical and corticothalamic models of the alpha rhythm
Source: PLoS Comput Biol. 2025 Apr 10;21(4):e1012926. doi: 10.1371/journal.pcbi.1012926 (PMC12064047; doi:10.1371/journal.pcbi.1012926)
Supplement: S4 Appendix — Fixed points behaviour and phase planes of JR and LW for different parameter sets when subjected to low input noise. (PDF) [file pcbi.1012926.s004.pdf]

S4 Appendix. Stability Analysis Low Noise JR and LW of Connectivity E-I parameters

In the main text, we presented the stability analysis of the E-I connectivity parameters with standard input. Our aim was to demonstrate the changes in this analysis when low noise input is introduced, highlighting the differences between the JR and LW models. Specifically, we showed that in the JR model, a Hopf bifurcation occurs at the alpha rhythm. In contrast, the alpha rhythm generated with LW model is noise-driven, as without noise, it behaves as a damped oscillator and reaches a fixed point instead of oscillating.

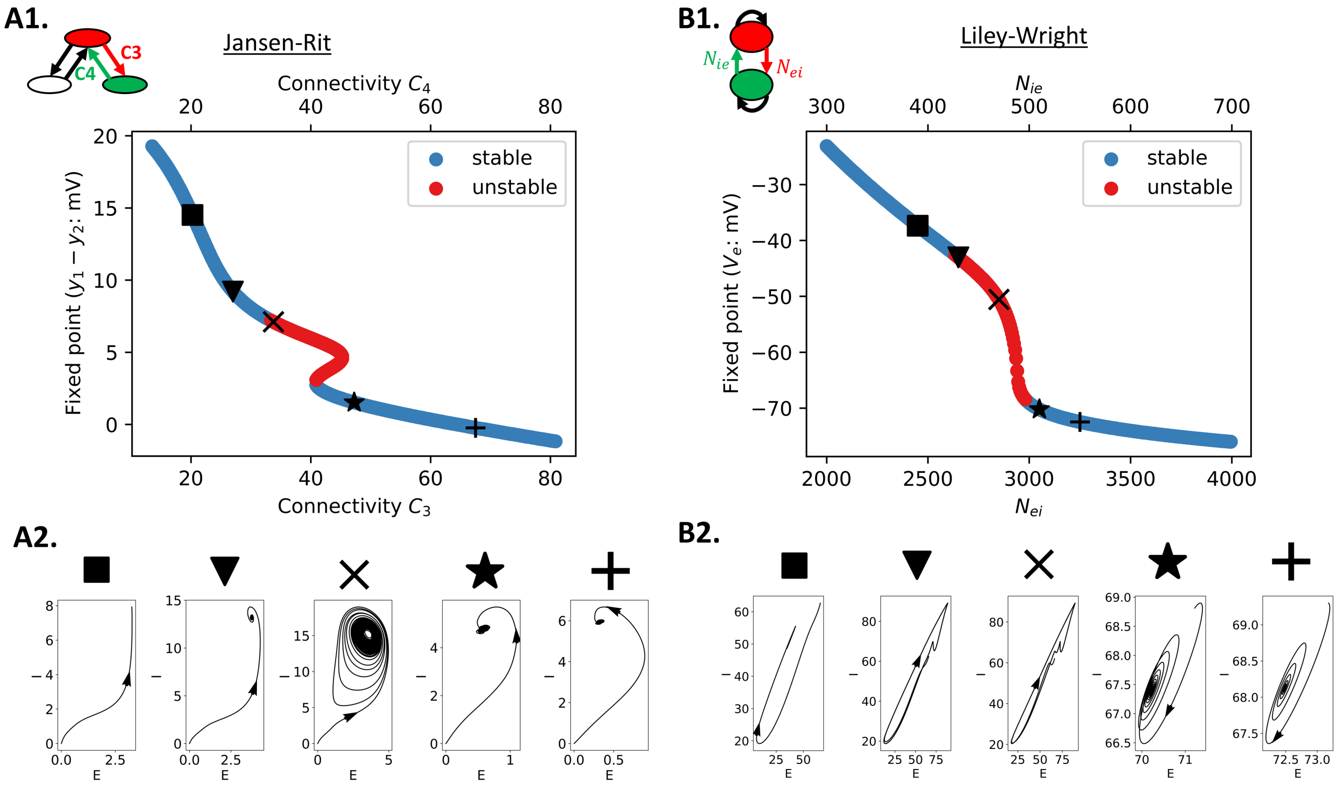

Fig A. ***Fixed points and corresponding phase planes of JR and LW at specific connectivity values with low noise input*** For the JR model, **A1** and **A2** correspond to the fixed points of JR with low noise, and the corresponding phase planes for specific connectivity values. Similarly to JR, the fixed points of LW are presented with low noise and the corresponding phase plane for specific connetivity values in **B1** and **B2**. Unstable fixed points are red, whereas stable fixed points are blue. The cross phase plan in JR correspond to the standard alpha rhythm parameters, whereas the star phase plane in LW correspond to the standard alpha parameters.
