# Supplementary material for: A comprehensive investigation of intracortical and corticothalamic models of the alpha rhythm
Source: PLoS Comput Biol. 2025 Apr 10;21(4):e1012926. doi: 10.1371/journal.pcbi.1012926 (PMC12064047; doi:10.1371/journal.pcbi.1012926)
Supplement: S5 Appendix — Includes the phase planes with the output voltage of the three populations under different connectivity values. (PDF) [file pcbi.1012926.s005.pdf]

S5 Appendix. Phase plane of JR in 3D

For the stability analyses in Fig. 11, we have only presented the phase plane with the pyramidal and inhibitory population output voltages. Considering the trajectory of the third excitatory neural population activity can provide a better understanding of the full picture, as presented in Fig. A.

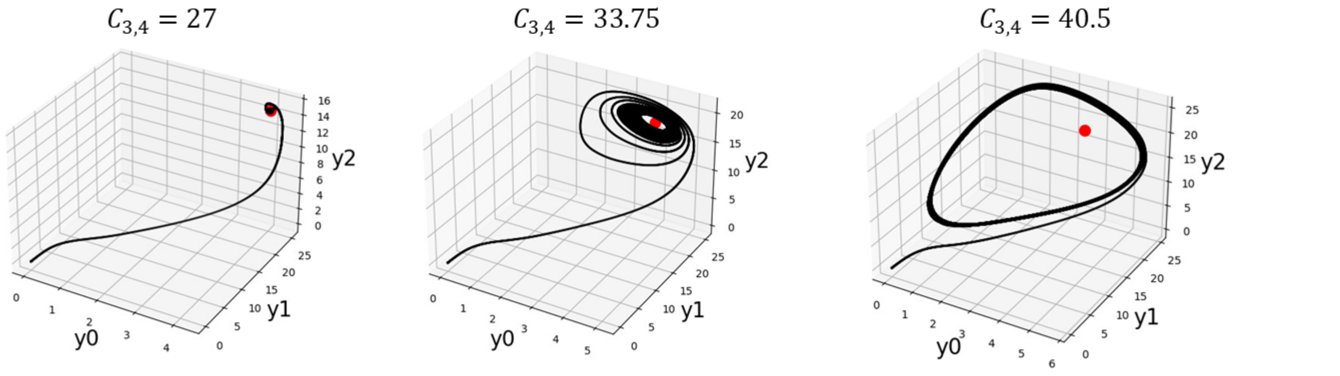

Fig A. ***Phase plane of JR for different E-I connectivity parameters.*** The trajectory of the three neural populations (with y_0, y_1, and y_2 corresponding to the output of the PSP block for the pyramidal cells, excitatory interneurons, and inhibitory interneurons, respectively) can be inferred by examining the stability of their respective fixed point (red). When $C_{3,4} = 27$, the fixed point is stable and no oscillations occur. For $C_{3,4} = 33.5$, the system enters a limit cycle with the oscillation frequency of alpha. Finally, when $C_{3,4} = 40.5$, the limit cycle widens and the frequency of oscillation is reduced.

As seen in our previous phase plane analysis, for specific connectivity parameters, the system either reaches a fixed point or enters a limit cycle defining the frequency of oscillation. The results closely resemble those in Fig. 11, implying that the dynamics primarily involve interactions between the pyramidal and inhibitory populations, with minimal contribution from the third population in this case.
