# Supplementary material for: A comprehensive investigation of intracortical and corticothalamic models of the alpha rhythm
Source: PLoS Comput Biol. 2025 Apr 10;21(4):e1012926. doi: 10.1371/journal.pcbi.1012926 (PMC12064047; doi:10.1371/journal.pcbi.1012926)
Supplement: S6 Appendix — Analyses the effect of connectivity parameters, namely the role of the self-inhibitory loop introduced in the MDF model compared to the JR model. (PDF) [file pcbi.1012926.s006.pdf]

S6 Appendix. Comparison of MDF and JR connectivity parameter spaces

By setting the parameters to be the same between JR and MDF, we compare the connectivity parameter space of the two models (Fig. A).

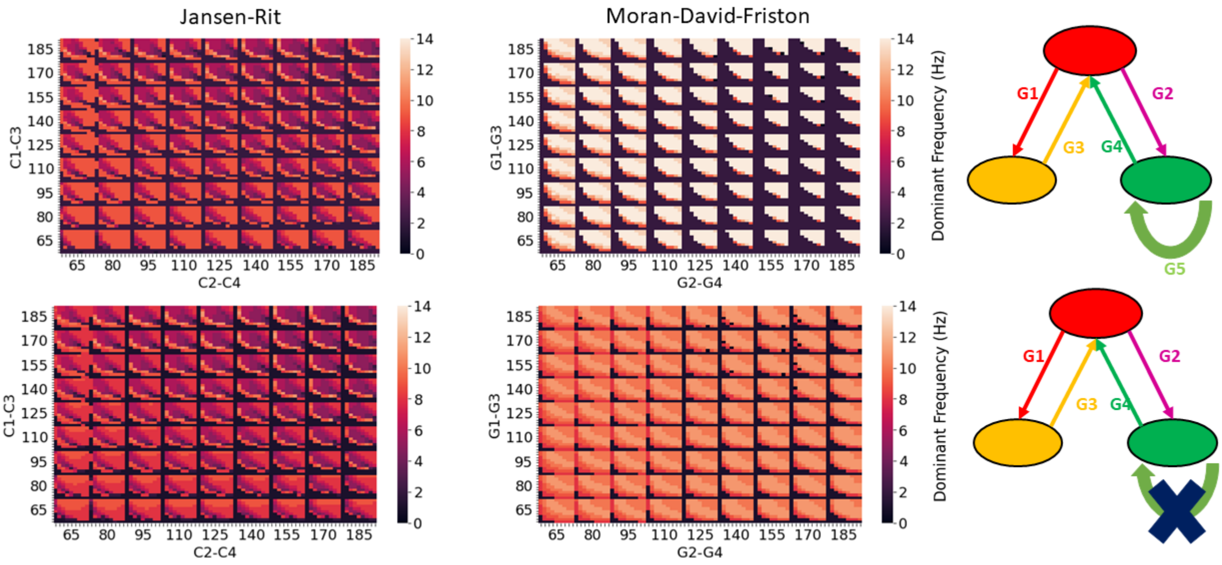

Fig A. ***Connection strength parameter spaces for JR and MDF with similar parameter settings.*** In the top, for MDF $\gamma_5 = 16$, and at the bottom $\gamma_5 = 0$. The general shape of the dynamics is very similar between the two, suggesting that the effects of connectivity are the same. However, MDF tends to generate oscillations of higher frequencies for identical connectivity parameter sets, even when the γ_5 connection is removed.

In the top row of Fig. A, we compare JR against MDF with the self-inhibitory connection. We observe a similar triangular boundary shape within which the system oscillates. However, MDF tends oscillate at higher frequency that exceed the alpha range (Fig. A, MDF top row, colors are brighter than JR). When the self-inhibitory connection is removed in MDF (Fig. A, MDF bottom row), the system now oscillates at the alpha frequency. It does not present lower frequencies, such as those in the JR model where we have slower oscillations. Thus, MDF seems to oscillate at higher frequencies than JR. Nonetheless, we observe that the two models share this similar triangular shape with non-oscillatory behavior when C_3 and C_4 are too low, suggesting similar global dynamics. The main conclusion drawn from this analysis is that the self-inhibitory connection introduced in MDF grants the model the ability to generate oscillations at a higher frequency than alpha, a more challenging capability compared to JR.
