# Supplementary material for: A comprehensive investigation of intracortical and corticothalamic models of the alpha rhythm
Source: PLoS Comput Biol. 2025 Apr 10;21(4):e1012926. doi: 10.1371/journal.pcbi.1012926 (PMC12064047; doi:10.1371/journal.pcbi.1012926)
Supplement: S7 Appendix — Presents the effects of the four connectivity parameters of JR simultaneously. (PDF) [file pcbi.1012926.s007.pdf]

S7 Appendix. 4D JR connectivity analysis

In the JR model, our focus was specifically on C_3 $(P \rightarrow I)$ and C_4 $(I \rightarrow P)$ as the E-I loop, but there is also the interaction between excitatory interneurons and pyramidal cells (C_1 $(P \rightarrow E)$ and C_2 $(E \rightarrow P)$) to consider. Typically, the ratio between these values is varied. By simulating time series for different values of C with the standard ratio values ($C_1 = C$, $C_2 = 0.8 * C$, $C_3 = 0.25 * C$ and $C_4 = 0.25 * C$), we can infer that increasing values of C lead to a decrease in the frequency of oscillation up to a certain point (Fig. A), which concurs with results from Jansen and Rit (1995) [1].

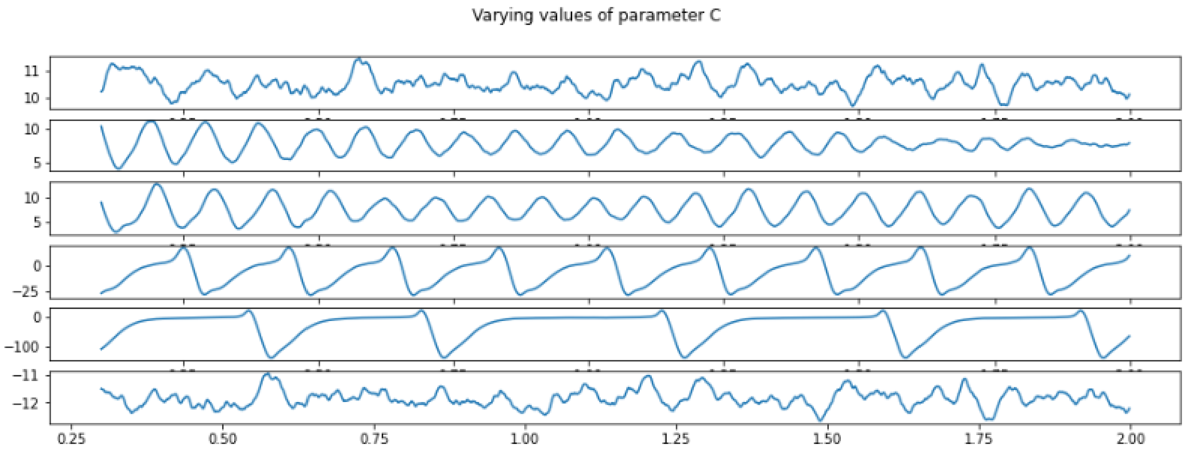

Fig A. ***Simulated time series of JR for different connectivity C values.*** From top to bottom: $C = 68$, $C = 128$, $C = 135$, $C = 270$, $C = 675$, $C = 1350$. As connectivity values increase, the frequency of oscillations decreases up to $C = 675$.

Changes in the frequency of oscillation as a function of connectivity ratios are presented in the form of 4D heatmaps in a 2D space (Fig. B). The general trend observed is that higher connectivity values result in slower oscillations, as expected.

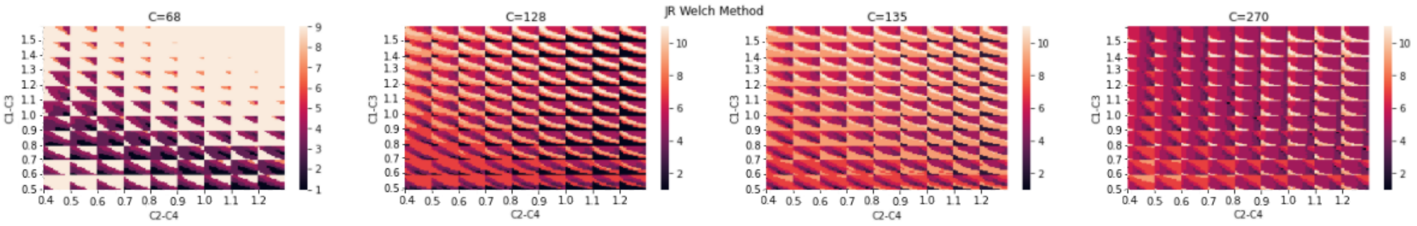

Fig B. ***Variation in the frequency of oscillation as a function of connection strength for JR for different*** C ***values.*** From left to right: $C = 68$, $C = 128$, $C = 135$ and $C = 270$. The outer axes $C_1 - C_2$ represent the excitatory loop, while the inner axes $C_3 - C_4$ represent the inhibitory loop. The results correlate with what is observed in the time series. The parameter space is obtained by changing the ratio of each connection (i.e: $C1 = 0.8$ corresponds to $C1 = 0.8 * C$)

To observe the different trends that emerge, we focused on the case where C equals 135 and investigated the different possible combinations of parameters on the outer and inner axes (Fig. C).

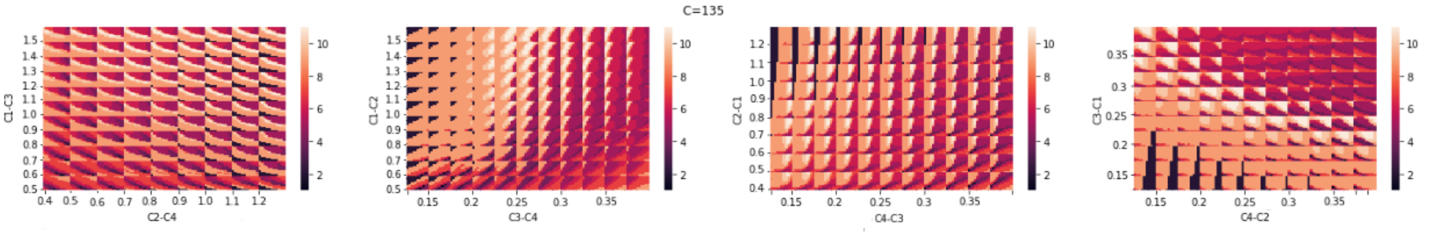

Fig C. ***Connection strength parameter spaces for Jansen-Rit in different combinations with C=135.*** Each combination reveals a distinct pattern, aiding in visualizing the relationships among all the connectivity parameters. From left to tight: 1) Outer axes $C_1 - C_2$ Inner axes $C_3 - C_4$; 2) Outer axes $C_1 - C_3$; Inner axes $C_2 - C_4$; 3) Outer axes $C_2 - C_3$; Inner axes $C_1 - C_3$; 4) Outer axes $C_3 - C_4$; Inner axes $C_1 - C_2$

Clear patterns emerge in two different cases. When C_3 $(P \to I)$ and C_4 $(I \to P)$ are on the outer axes, a continuous change in the frequency of oscillation is observed. Similarly, when comparing C_1 $(P \to E)$ against C_3 $(P \to I)$, a concrete pattern is evident, with more pronounced changes in the frequency of oscillation when C_3 is altered. These results reinforce the idea that the main loop influencing the frequency of oscillation is the interaction between the pyramidal and inhibitory populations, raising the question of whether adding an additional excitatory population is truly necessary, even though it would be more biologically realistic.

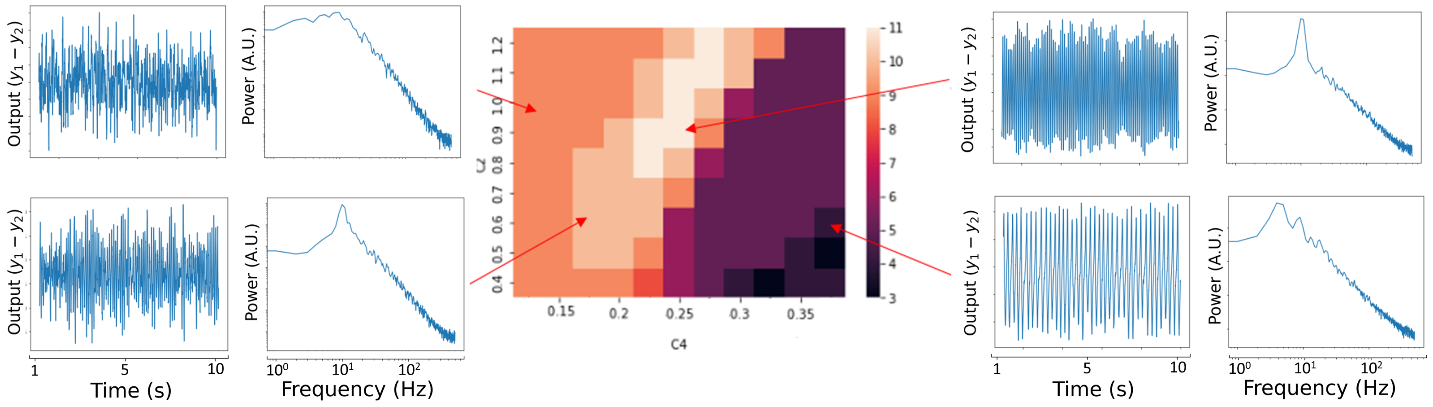

Fig D. ***Connection strength parameter space for C_2 $(E \to P)$ and C_4 $(I \to P)$ in JR.*** Higher values of C_4 lead to a decrease in rhythmic oscillations. The highest frequency of oscillation occurs when C_4 is at a ratio of 0.25, and C_2 is around 1.0. If C_4 is too low, a very noisy signal is generated.

References

[1] Jansen BH, Rit VG. Electroencephalogram and visual evoked potential generation in a mathematical model of coupled cortical columns. Biological cybernetics. 1995;73(4):357–366.
