# Supplementary material for: A comprehensive investigation of intracortical and corticothalamic models of the alpha rhythm
Source: PLoS Comput Biol. 2025 Apr 10;21(4):e1012926. doi: 10.1371/journal.pcbi.1012926 (PMC12064047; doi:10.1371/journal.pcbi.1012926)
Supplement: S8 Appendix — Reduces the 5-dimensional connectivity parameter space to a 3-dimensional representation, to assess the contributions from the circuit loops. (PDF) [file pcbi.1012926.s008.pdf]

S8 Appendix. 3D parameter space with MDF

We simplified the 5-dimensional connection parameter space into a 3-dimensional representation for the MDF model, using its linearized version. Stability is assessed by looking at the system's poles within the transfer function of the system. The aim was to establish a parallel with the 3D 'xyz' corticocortical/corticothalamic/intrathalamic lumped gains reduced parameter space discussed in a number of studies using the RRW model (although for reasons of space we have not focused on that aspect of RRW in the present paper [1–5]), and determine the effects of the loops on the dynamics of the MDF model.

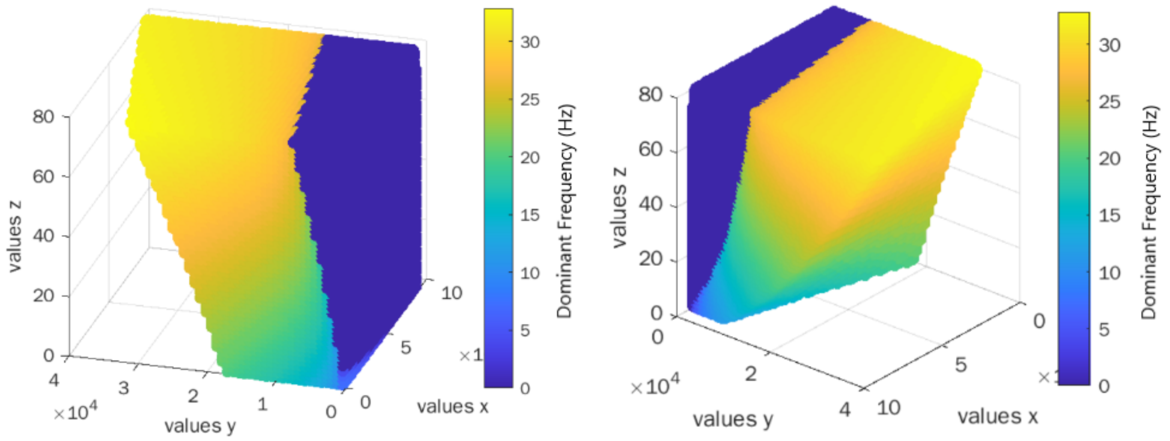

Fig A. ***Visualization of dynamical regimes of the MDF model in a 3D setting using the linearized expression.*** The x-axis corresponds to effect of the excitatory loop ($\gamma_1 * \gamma_2$); the y-axis represents the effect of the inhibitory loop ($\gamma_3 * \gamma_4$); and the z-axis is the effect of the self-inhibitory loop (γ_5). As γ_5 values increase, the system tends to oscillate at a higher frequency.

The aim here is to easily visualize the regions of stability and dynamics as a function of the 'loops', rather than a single connectivity parameter. As expected, with the increase in the self-inhibitory connection (z-axis), the dominant frequency of oscillation gradually shifts from theta to alpha and then to the beta range.

References

[1] Robinson P, Rennie C, Rowe D. Dynamics of large-scale brain activity in normal arousal states and epileptic seizures. Physical Review E. 2002;65(4):041924.

[2] Robinson PA, Rennie C, Rowe DL, O'Connor S, Gordon, E. Multiscale brain modelling. Philosophical Transactions of the Royal Society B: Biological Sciences. 2005;360(1457):1043–1050.

[3] Roberts J, Robinson P. Corticothalamic dynamics: structure of parameter space, spectra, instabilities, and reduced model. Physical Review E. 2012;85(1):011910.

[4] Breakspear M, Roberts JA, Terry JR, Rodrigues S, Mahant N, Robinson PA. A unifying explanation of primary generalized seizures through nonlinear brain modeling and bifurcation analysis. Cerebral Cortex. 2006;16(9):1296–1313.

[5] Abeysuriya R, Rennie C, Robinson P. Physiologically based arousal state estimation and dynamics. Journal of Neuroscience Methods. 2015;253:55–69.
