# Supplementary material for: A comprehensive investigation of intracortical and corticothalamic models of the alpha rhythm
Source: PLoS Comput Biol. 2025 Apr 10;21(4):e1012926. doi: 10.1371/journal.pcbi.1012926 (PMC12064047; doi:10.1371/journal.pcbi.1012926)
Supplement: S9 Appendix — Provides the complete set of equations for the models, along with tables detailing the parameter descriptions and their standard values. (PDF) [file pcbi.1012926.s009.pdf]

S9 Appendix. Full Model Equations

Partial and diagrammatic presentations of the differential equations for each of the four models are given in Figs 4-7. In this Appendix, we provide the complete differential equations for each model, as well as tables describing the model parameters and state variables.

Jansen-Rit model equations

The differential equations for the JR model are

$$\dot{y}_0(t) = y_3(t) \tag{1}$$
$$\dot{y}_3(t) = AaS[y_1(t) - y_2(t)] - 2ay_3(t) - a^2 y_0(t) \tag{2}$$
$$\dot{y}_1(t) = y_4(t) \tag{3}$$
$$\dot{y}_4(t) = Aa(p(t) + C_2 S[C_1 y_0(t)]) - 2ay_4(t) - a^2 y_1(t) \tag{4}$$
$$\dot{y}_2(t) = y_5(t) \tag{5}$$
$$\dot{y}_5(t) = BbC_4 S[C_3 y_0] - 2by_5(t) - b^2 y_2(t) \tag{6}$$

Here and in the rest of this paper we have maintained the same notation as in Jansen and Rit [1] where y_0, y_1, and y_2 correspond to the outputs of the pyramidal, excitatory, and inhibitory PSP block, respectively. $p(t)$ represents the external input applied to the system, usually noise. A and B define the maximum amplitude of excitatory and inhibitory PSP, respectively. a and b represent the collective effect of the inverse of the time constant of the passive membrane and the entirety of the spatially dispersed delays within the dendritic network for the excitatory and inhibitory populations, respectively. C_1 to C_4 are the connectivity constants.

For the connectivity parameters, we wanted to mention that C_1 and C_3 slightly differ from C_2 and C_4 in the mathematical expression. The JR model assumes equal synaptic input from the pyramidal cell population to the other two populations, setting these constants to 1. In contrast, the synaptic coefficients at the excitatory and inhibitory dendrites are varied (corresponding to C_1 ($P \to E$) and C_3 ($P \to I$)). Conversely, for pyramidal cells, the synaptic coefficients at their dendrites remain fixed (1 and -1 for excitatory and inhibitory interneurons, respectively), and excitatory and inhibitory neurons synapse onto pyramidal cells differently (represented by C_2 ($E \to P$) and C_4 ($I \to P$). Therefore, C_1 and C_3 function as synaptic coefficients, while C_2 and C_4 serve as connectivity constants, as illustrated in the detailed schematic. Mathematically, this means that C_1 and C_3 are applied within the nonlinear function, while C_2 and C_4 are applied outside. However, in practical terms, all these parameters are described as connectivity parameters and can be considered analogous and interrelated. Furthermore, all the values are scaled by a global connectivity parameter. See Cook et al. 2021 [2] for a further explanation of this nuanced aspect of the JR model system.

Symbol	Description	Value
e_0	Firing rate at threshold	2.5 $\mathrm{s}^{(-1)}$
V_0	Firing threshold	6 mV
r	Slope reflecting the variance of firing thresholds within the population	0.56 $\mathrm{mV}^{(-1)}$
A	Maximum amplitude of excitatory PSP (EPSP)	3.25 mV
B	Maximum amplitude of inhibitory PSP (IPSP)	22 mV
a and b	Lumped representation of the sum of the reciprocal of the time constant of passive membrane and all other spatially distributed delays in the dendritic network	a = 100 $\mathrm{s}^{(-1)}$<br>b = 50 $s^{(-1)}$
C_1	Connectivity constant: Represents the number of synapses made by the feed forward neurons to the dendrites of the excitatory feedback loop	$C = C_1$<br>135
C_2	Connectivity constant: Proportional to the number of synapses made by the excitatory feedback loop to the dendrites of the feedforward neurons	$C_2 = 0.8C$
C_3	Connectivity constant: number of synapses made by the feedforward neurons to the dendrites of the inhibitory feedback loop	$C_3 = 0.25C$
C_4	Connectivity constant: Proportional to the number of synapses made by the inhibitory feedback loop to the dendrites of the feedforward neurons	$C_4 = 0.25C$
P(t)	External pulse density consisting of activity originating from adjacent and more distant cortical columns and from subcortical structures (e.g. thalamus)	Uniform noise (or normal, constant)

Table A. ***JR parameters with biological descriptions and corresponding values to generate alpha rhythm***

Moran-David-Friston model equations

The form of the differential equations for the MDF model are

$$\dot{\nu}_1 = i_1 \tag{7}$$

$$\dot{i}_1 = \kappa_e H_e(\gamma_1 S(\nu_6 - a) + u) - 2\kappa_e i_1 - \kappa_e^2 \nu_1 \tag{8}$$

$$\dot{\nu}_2 = i_2 \tag{9}$$

$$\dot{i}_2 = \kappa_e H_e \gamma_2 S(\nu_1) - 2\kappa_e i_2 - \kappa_e^2 \nu_2 \tag{10}$$

$$\dot{\nu}_3 = i_3 \tag{11}$$

$$\dot{i}_3 = \kappa_i H_i \gamma_4 S(\nu_7) - 2\kappa_i i_3 - \kappa_i^2 \nu_3 \tag{12}$$

$$\dot{\nu}_6 = i_2 - i_3 \quad (13)$$

$$\dot{\nu}_4 = i_4 \quad (14)$$

$$\dot{i}_4 = \kappa_e H_e \gamma_3 S(\nu_6) - 2\kappa_e i_4 - \kappa_e^2 \nu_4 \quad (15)$$

$$\dot{\nu}_5 = i_5 \quad (16)$$

$$\dot{i}_5 = \kappa_i H_i \gamma_5 S(\nu_7) - 2\kappa_i i_5 - \kappa_i^2 \nu_5 \quad (17)$$

$$\dot{\nu}_7 = i_4 - i_5 \quad (18)$$

The v_i values represent the membrane potential of the subpopulations and i_i denoting their current. Specifically, v_1 and i_1 describe the excitatory interneurons, $v_{2,3,6}$ and $i_{2,3}$ the pyramidal cells, and finally $v_{4,5,7}$ and $i_{4,5}$ the inhibitory interneurons. The γ_i values are the connection strengths between the populations. H_e and κ_e are the maximum amplitude and the rate constant associated with EPSP, respectively. Similarly, H_i and κ_i represent the same parameters for the IPSP.

Symbol	Description	Value
ρ_1	For shape of sigmoid: Can straighten more or less the slope	2
ρ_2	For position of sigmoid: Can shift the curve right or left	1
H_e	Maximum amplitude of excitatory PSP (EPSP)	10 mV
H_i	Maximum amplitude of inhibitory PSP (IPSP)	22 mV
κ_e and κ_i	Lumped representation of the sum of the rate constants of passive membrane and other spatially distributed delays in the dendritic tree	$\kappa_e = 250$ s$^{(-1)}$ $\kappa_i = 62.5$ s$^{(-1)}$
γ_1	Coupling strength: Between pyramidal cells and macrocolumn u (in excitatory spiny cells in granular layer)	128
γ_2	Coupling strength: Between excitatory spiny cells in granular layer and pyramidal cells	128
γ_3	Coupling strength: Between pyramidal cells (excitatory) and inhibitory interneurons	64
γ_4	Coupling strength: Between inhibitory interneurons and pyramidal cells	64
γ_5	Coupling strength: Inhibitory-Inhibitory coupling (recurrent connection)	1

Table B. ***MDF parameters with biological descriptions and corresponding values to generate alpha rhythm***

Liley-Wright model equations

For the LW model, the differential equations are

$$
\begin{aligned}
\tau_e \dot{V}_e(t) &= V_e^{rest} - V_e(t) + \psi_{ee}(V_e(t))I_{ee}(t) + \psi_{ie}(V_e(t))I_{ie}(t) && (19)\\
\tau_i \dot{V}_i(t) &= V_i^{rest} - V_i(t) + \psi_{ei}(V_i(t))I_{ei}(t) + \psi_{ii}(V_i(t))I_{ii}(t) && (20)\\
\dot{I}_{ee} &= U_{ee} && (21)\\
\dot{U}_{ee} &= -2\gamma_e U_{ee}(t) - \gamma_e^2 I_{ee}(t) + \Gamma_e \gamma_e e(N_{ee}^{\beta} S(V_e(t)) + p_{ee}(t)) && (22)\\
\dot{I}_{ei} &= U_{ei} && (23)\\
\dot{U}_{ei} &= -2\gamma_e U_{ei}(t) - \gamma_e^2 I_{ei}(t) + \Gamma_e \gamma_e e(N_{ei}^{\beta} S(V_e(t)) + p_{ei}(t)) && (24)\\
\dot{I}_{ie} &= U_{ie} && (25)\\
\dot{U}_{ie} &= -2\gamma_i U_{ie}(t) - \gamma_i^2 I_{ie}(t) + \Gamma_i \gamma_i e(N_{ie}^{\beta} S(V_i(t))) && (26)\\
\dot{I}_{ii} &= U_{ii} && (27)\\
\dot{U}_{ii} &= -2\gamma_i U_{ii}(t) - \gamma_i^2 I_{ii}(t) + \Gamma_i \gamma_i e(N_{ii}^{\beta} S(V_i(t))) && (28)
\end{aligned}
$$

N_{xx} are the inter- and intra-connectivities between the two populations. p_{ei} and p_{ee} are the external inputs. I_{xx} are the postsynaptic potentials, and V_{xx} are the soma membrane potentials. $\Gamma_{e,i}$ and $\gamma_{e,i}$ are the peak amplitude and rate constant PSPs for excitatory and inhibitory population, respectively. The model also includes passive membrane time constants represented by $\tau_{e,i}$, mean resting membrane potentials $V_{e,i}^r$, and mean equilibrium potentials $V_{e,i}^{eq}$.

Symbol	Description	Value
$S_{(e,i)}^{max}$	Excitatory/Inhibitory population mean maximal firing rates	500, 500 $s^{(-1)}$
$\mu_{(e,i)}$	Excitatory/Inhibitory population thresholds (spike threshold)	-50, -50 mV
$\sigma_{(e,i)}$	Standard deviation for spike-threshold in excitatory/inhibitory population	5, 5 mV
Γ_e	Excitatory postsynaptic potential peak amplitude (at the site of synaptic activation)	0.71 mV
Γ_i	Inhibitory postsynaptic potential peak amplitude (at the siyte of synaptic activation)	0.71 mV
$\gamma_{(e,i)}$	Excitatory/Inhibitory postsynaptic potential rate constant	300, 65 $s^{(-1)}$
$\tau_{(e,i)}$	Passive membrane decay time constant	0.094, 0.042 s
$V_{(e,i)}^r$	Mean resting membrane potential	-70, -70 mV
$V_{(e,i)}^{eq}$	Mean equilibrium potential associated with excitation or inhibition	45, -90 mV
$N_{(ee,ei)}^{\beta}$	Total number of connections that a cell of type e, i receives from excitatory cells via intra-cortical fibres (Weight connections)	3000, 3000
$N_{(ie,ii)}^{\beta}$	Total number of connections that a cell of type e,i receives from inhibitory cells via intra-cortical connections (Weight connections)	500, 500
$p_{(ee,ei)}$	Excitatory extra-cortical input to excitatory, inhibitory cells	3.46, 5.07$s^{(-1)}$
$p_{(ie,ii)}$	Inhibitory extra-cortical input to excitatory, inhibitory cells	0, 0 $s^{(-1)}$

Table C. ***LW parameters with biological descriptions and values to generate alpha rhythm***

Robinson-Rennie-Wright model equations

Finally, the differential equations of the RRW are as follows

$$\frac{dV_e}{dt} = \dot{V}_e \quad (29)$$

$$\frac{d\dot{V}_e}{dt} = \alpha\beta[\nu_{ee}\phi_e + \nu_{ei}S(V_e) + \nu_{es}S(V_s(t - t_0/2)) - (\frac{1}{\alpha} + \frac{1}{\beta})\dot{V}_e - V_e] \quad (30)$$

$$\frac{dV_s}{dt} = \dot{V}_s \quad (31)$$

$$\frac{d\dot{V}_s}{dt} = \alpha\beta[\nu_{se}\phi_e(t - t_0/2) + \nu_{sr}S_r(V_r) + \nu_{sn}\phi_n - (\frac{1}{\alpha} + \frac{1}{\beta})\dot{V}_s - V_s] \quad (32)$$

$$\frac{dV_r}{dt} = \dot{V}_r \quad (33)$$

$$\frac{d\dot{V}_r}{dt} = \alpha\beta[\nu_{re}\phi_e(t - t_0/2) + \nu_{rs}S(V_s) - (\frac{1}{\alpha} + \frac{1}{\beta})\dot{V}_e - V_r] \quad (34)$$

$$\frac{d\phi_e}{dt} = \dot{\phi}_e \quad (35)$$

$$\frac{d\dot{\phi}_e}{dt} = \gamma_e^2[S(V_e) - \frac{2}{\gamma_e}\dot{\phi}_e - \phi_e] \quad (36)$$

with V_e, V_r, and V_s representing the potential of the cortical population, the reticular nucleus, and the relay nuclei, respectively. ν_{xx} denote the connection strengths parameters. α and β refer to the decay and rise time of the impulse response, representing the dendritic rate. t_0 is the conduction delay between thalamic and cortical projections. Finally, γ_e stands for the cortical damping rate, which is exclusively applied to the cortical population. This final differential equation for determining ϕ_e is related to the PDE damped wave equation, used to consider spatial variations [3]. However, in the case of spatial uniformity, the wave equation simplifies to an ODE [4].

Symbol	Description	Value
Q_{max}	Maximum attainable firing rate of individual neurons	340 $\mathrm{s}^{(-1)}$
$\sigma'\pi\sqrt{3}$	Standard deviation of the threshold distribution in the neural population	$3.8{*}\pi\sqrt{3} \approx$ 5.9 mV
θ	Mean firing threshold	12.92 mV
γ_e	Cortical damping rate (Axonal velocity/Range)	116 $s^{(-1)}$
$1/\alpha$	Decay time (of impulse response, dendritic rate)	83.33 s^{-1}
$1/\beta$	Rise time (of impulse response, dendritic rate)	769.23 s^{-1}
t_0	Corticothalamic loop delay (Loop distance/Axonal velocity = conduction delay through thalamic nuclei and projections)	80 ms
v_{ee}	$N_{ee}s_{ee}$: Mean number of synapses X strength of the response to a unit signal	3.03 mVs
$-v_{ei}$	$-N_{ei}s_{ei}$	6.00 mVs
v_{es}	$N_{es}s_{es}$	2.06 mVs
v_{se}	$N_{se}s_{se}$	2.18 mVs
$-v_{sr}$	$-N_{sr}s_{sr}$	0.83 mVs
v_{re}	$N_{re}s_{re}$	0.33 mVs
v_{rs}	$N_{rs}s_{rs}$	0.03 mVs
v_{sn}	$N_{sn}s_{sn}$	0.98 mVs

Table D. ***RRW parameters with biological descriptions and values to generate alpha rhythm***

References

[1] Jansen BH, Rit VG. Electroencephalogram and visual evoked potential generation in a mathematical model of coupled cortical columns. Biological cybernetics. 1995;73(4):357–366.

[2] Cook BJ, Peterson AD, Woldman W, Terry JR. Neural Field Models: historical perspectives and recent advances. arXiv preprint arXiv:210310554. 2021;.

[3] Robinson PA, Rennie CJ, Wright JJ. Propagation and stability of waves of electrical activity in the cerebral cortex. Physical Review E. 1997;56(1):826.

[4] Zhao X, Robinson PA. Generalized seizures in a neural field model with bursting dynamics. Journal of computational neuroscience. 2015;39(2):197–216.
