# Supplementary material for: A comprehensive investigation of intracortical and corticothalamic models of the alpha rhythm
Source: PLoS Comput Biol. 2025 Apr 10;21(4):e1012926. doi: 10.1371/journal.pcbi.1012926 (PMC12064047; doi:10.1371/journal.pcbi.1012926)
Supplement: S10 Appendix — Offers supplementary information on the early development of NPMs and other existing theories of alpha rhythmogenesis. (PDF) [file pcbi.1012926.s010.pdf]

10 Appendix. Additional background literature

For interested readers, we provide here some further background literature on early history of NPMs and other alpha theories:

Tracing the roots of NPMs: early history

The notion of neural *masses* was introduced in various forms during the 1950s and 1960s [1, 2], and consolidated in the 1970s primarily through the highly influential work of Freeman, Wilson & Cowan, Amari, and Nunez. It was Freeman who originally used the term 'neural mass action model' [3–5], articulating many of the neurobiological and mathematical fundamentals as they are understood today in a wide-reaching monograph on the subject [5]. Here, Freeman also develops the theory of 'K-sets' which are based on a hierarchy of interacting sets of neural populations or masses, and used to model neural population dynamics with ordinary differential equations (ODEs) to simulate mesoscopic local field potentials [6]. The levels are designated as K0, KI, KII, and KIII, with the K0 set corresponding to a model characterized by non-interactive collections of neurons with globally common inputs and outputs, KI to pairs of interacting K0 sets, and so on. Freeman's research on the olfactory bulb and prepyriform cortex of cats and rabbits [5, 7] provides valuable experimental data that has been used to define mathematical formulations and parameter settings in many NMMs, which is further discussed in section 3.2.4. Furthermore, Freeman's contributions on the use of the sigmoidal operator for mapping membrane potential to firing rate remains a critical component of many NMMs, the validity of which will be elaborated on in section 4.2. Even though Freeman coined the term neural masses and laid much of the groundwork, many of the core mathematical principles of NMMs were first proposed in the work of Wilson & Cowan (WC; [8]), which itself builds upon earlier work by Beurle (1956) [1]. WC's implementation introduced and solidified an approach to modelling neural dynamics and brain function. This approach consists of analyzing the collective properties of a large number of neurons using methods from statistical mechanics rooted in the mean-field framework [9, 10]. By omitting potential spatial arrangement of synaptic connections, their model offers a minimalistic NMM representation that has been leveraged to develop several simple yet biophysically plausible models (eg [11, 12]). As shown in Fig. A, the canonical WC model consists of two neural masses with one excitatory and one inhibitory population [8, 12]. Two nonlinear ODEs describe the dynamics of those two synaptically coupled populations in the neocortex [13, 14]. The WC system is thus a coarse-grained description of the overall activity and mesoscale neuronal network structure of a patch of (usually cortical) tissue, as is typical of NPMs. By varying the connectivity strength and the input strength to each population, it is possible to generate a diversity of dynamical behaviors that are characteristic of observed activity in the brain, such as multistability, oscillations, traveling waves, and spatial patterns [11].

A simplified version of the WC equations shown in Fig. A has been previously implemented by Abeysuriya et al. (2018) [15] in a network of neural masses to generate alpha oscillations. These two populations are described as follows:

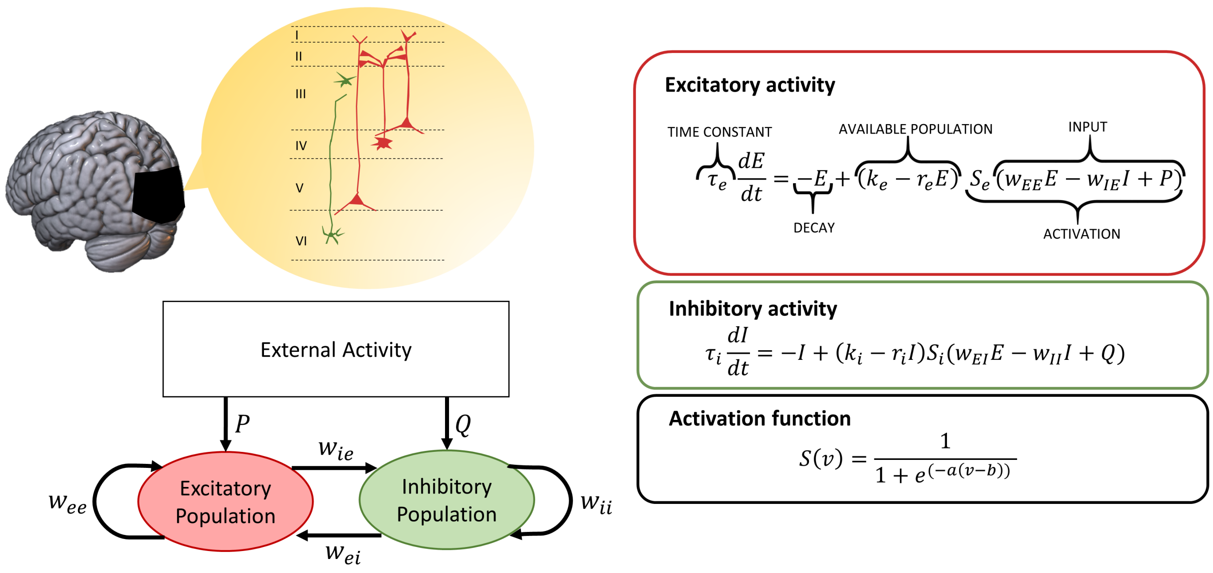

Fig A. ***Wilson-Cowan model topography and mathematical expression.*** The model aims to represent a cortical column within the brain, consisting of an excitatory and an inhibitory population. These two connected populations each have a self-connection and external activity as input. Dynamics are expressed with nonlinear ordinary differential equations which are shown on the right for each neural population. Nonlinearity is introduced with the sigmoidal operator corresponding to the activation function.

$$\tau_e \frac{dE(t)}{dt} = -E(t) + S(w_{ee}E(t) + w_{ie}I(t) + P + \epsilon(t)) \tag{1}$$

$$\tau_i \frac{dI(t)}{dt} = -I(t) + S(w_{ei}E(t) + \epsilon(t)) \tag{2}$$

where E and I represent the activity of the excitatory and inhibitory neural populations in the form of mean firing rates, $\tau_{e/i}$ are the excitatory/inhibitory time constants, w_{ab} are the local connection strengths from population a to population b, P is a constant external input to the excitatory neural population, and ϵ is a noise signal added to the system. The studied NMMs share similar parameters, with some variations such as the use of membrane potential instead of firing rates as the state variable, and the concatenation of the external input and noise term into a single variable.

Concurrently to WC and Freeman, Lopes da Silva and colleagues developed a point-process model of EEG alpha rhythm generated with a corticothalamic loop [16]. Specifically, these authors proposed a negative feedback loop between excitatory thalamocortical relay cells and inhibitory thalamic reticular neurons as the basis for generating certain brain rhythms, in a manner similar to the interacting E and I populations in the WC model. By applying linear systems analysis to investigate the influence of physiological parameters on neural periodic patterns, they established a novel approach to studying oscillatory dynamics in theoretical neuroscience that relied on analytical power spectra. The Lopes da Silva model had a substantial impact on subsequent corticothalamic models and linear analysis tools [17, 18].

A few years later, Zetterberg et al. (1978) [19] built an extension of the model by adding a second cortical excitatory population in order to separately account for pyramidal cells and

excitatory interneurons. Their work was then reprised and further popularized by Jansen and Rit (1995) [20]. In the JR model, each neural population is described in two steps: a transformation of the incoming average pulse density of action potentials into an average postsynaptic membrane potential, followed by a sigmoidal function to perform the inverse conversion. Over the years, several extended versions of JR have been proposed [21–24], - including Moran et al., where they focused on steady-state spectral responses with a linearized approximation of the model [25]. Contemporaneous with these early conceptualizations and formulations of NMMs in the 1970s was the introduction of NFMs by Amari, Wilson & Cowan, Nunez, and others. The 'brain wave equation' model of [26] is particularly important here as it was the first to attempt to describe neural activity across the entire cerebral cortex with an evolution in both time and space. This work was a major influence for several macroscale NFM formulations in the 1990s [27–29]. The latter of these which was then extended in 2001 to include the thalamus, and subsequently used to investigate a wide range of brain states including sleep [30, 31], epileptic seizures [32, 33], evoked responses [34], functional connectivity [35], and alpha rhythms [30, 36].

For a more detailed timeline and review on the development of NPMs and whole brain modelling in general, we refer the reader to Griffiths et al. (2022) [37] and Chow et al. (2020) [10]. The early mathematical models reviewed there and above laid the groundwork for most NPM formulations used in theoretical neuroscience today. In particular, they form the basis for the four most widely studied models of the EEG alpha rhythm - Jansen-Rit (JR), Moran-David-Friston (MDF), Liley-Wright (LW) and Robinson-Rennie-Wright (RRW).

Pacemaker vs. Local Network vs. Global Network Alpha theories

As indicated in the main text, the theories of alpha in the literature can be grouped under three categories: *pacemaker*, *local network*, and *global network* theories. For reasons of space we discuss only the second of these in this article. The following provide some further notes and references for the interested reader on the other two. The pacemaker theory suggests that intrinsic alpha oscillations are generated either in the thalamus, driven by pulvinar or and/or the lateral geniculate nucleus [38–40] or in the cortex, originating from the pyramidal cells located in layer V [41–43]. However, pacemaker theories in general suffer from several severe limitations (see Nunez (2006) [44] for an extensive discussion of this). For instance, pacemaker cells such as putative thalamic nuclei, if they exist, would have to function in a relatively autonomous fashion, having a highly restricted input from other oscillatory brain regions - a notion that has been critically questioned on anatomical grounds [45, 46]. Additionally, there are certain global EEG phenomena that remain unexplained, including the relative frequencies of major rhythms and sleep-wave variations. The second category, 'local network' theories, propose that alpha rhythms are produced by interactions between excitatory and inhibitory neural populations with dendritic response functions and saturating nonlinearities [47]. Finally, 'global network' theories posit that alpha rhythms are generated by large-scale networks rather than local circuits within a localized brain region. By disregarding complex dendritic response functions and finite intracortical propagation, models with a primary emphasis on global dynamics rely heavily on

the propagation delays between distant anatomical structures to shape their dynamics [47–49].

References

[1] Beurle RL. Properties of a mass of cells capable of regenerating pulses. Philosophical Transactions of the Royal Society of London Series B, Biological Sciences. 1956; p. 55–94.

[2] Griffith JS. A field theory of neural nets: I: Derivation of field equations. The bulletin of mathematical biophysics. 1963;25:111–120.

[3] Freeman WJ. Linear analysis of the dynamics of neural masses. Annual review of biophysics and bioengineering. 1972;1(1):225–256.

[4] Freeman WJ. Waves, pulses, and the theory of neural masses. Progress in theoretical biology. 1972;2(1):1–10.

[5] Freeman WJ. Mass action in the nervous system. vol. 2004. Citeseer; 1975.

[6] Deschle N, Ignacio Gossn J, Tewarie P, Schelter B, Daffertshofer A. On the validity of neural mass models. Frontiers in computational neuroscience. 2021; p. 118.

[7] Freeman WJ. Nonlinear gain mediating cortical stimulus-response relations. Biological cybernetics. 1979;33(4):237–247.

[8] Wilson HR, Cowan JD. Excitatory and inhibitory interactions in localized populations of model neurons. Biophysical journal. 1972;12(1):1–24.

[9] Destexhe A, Sejnowski TJ. The Wilson–Cowan model, 36 years later. Biological cybernetics. 2009;101(1):1–2.

[10] Chow CC, Karimipanah Y. Before and beyond the Wilson–Cowan equations. Journal of neurophysiology. 2020;123(5):1645–1656.

[11] Kilpatrick ZP. In: Jaeger D, Jung R, editors. Wilson-Cowan Model. New York, NY: Springer New York; 2013. p. 1–5. Available from: `https://doi.org/10.1007/978-1-4614-7320-6_80-1`.

[12] Sanz-Leon P, Knock SA, Spiegler A, Jirsa VK. Mathematical framework for large-scale brain network modeling in The Virtual Brain. Neuroimage. 2015;111:385–430.

[13] Nakagawa TT, Woolrich M, Luckhoo H, Joensson M, Mohseni H, Kringelbach ML, et al. How delays matter in an oscillatory whole-brain spiking-neuron network model for MEG alpha-rhythms at rest. Neuroimage. 2014;87:383–394.

[14] Cowan JD, Neuman J, van Drongelen W. Wilson–Cowan equations for neocortical dynamics. The Journal of Mathematical Neuroscience. 2016;6(1):1–24.

[15] Abeysuriya RG, Hadida J, Sotiropoulos SN, Jbabdi S, Becker R, Hunt BA, et al. A biophysical model of dynamic balancing of excitation and inhibition in fast oscillatory large-scale networks. PLoS computational biology. 2018;14(2):e1006007.

[16] Lopes da Silva F, Hoeks A, Smits H, Zetterberg L. Model of brain rhythmic activity. Kybernetik. 1974;15(1):27–37.

[17] Cona F, Lacanna M, Ursino M. A thalamo-cortical neural mass model for the simulation of brain rhythms during sleep. Journal of Computational Neuroscience. 2014;37(1):125–148.

[18] Bhattacharya BS, Coyle D, Maguire LP. A thalamo–cortico–thalamic neural mass model to study alpha rhythms in Alzheimer's disease. Neural networks. 2011;24(6):631–645.

[19] Zetterberg LH, Kristiansson L, Mossberg K. Performance of a model for a local neuron population. Biological cybernetics. 1978;31(1):15–26.

[20] Jansen BH, Rit VG. Electroencephalogram and visual evoked potential generation in a mathematical model of coupled cortical columns. Biological cybernetics. 1995;73(4):357–366.

[21] Wendling F, Bellanger JJ, Bartolomei F, Chauvel P. Relevance of nonlinear lumped-parameter models in the analysis of depth-EEG epileptic signals. Biological cybernetics. 2000;83(4):367–378.

[22] David O, Friston KJ. A neural mass model for MEG/EEG:: coupling and neuronal dynamics. NeuroImage. 2003;20(3):1743–1755.

[23] Zavaglia M, Astolfi L, Babiloni F, Ursino M. A neural mass model for the simulation of cortical activity estimated from high resolution EEG during cognitive or motor tasks. Journal of neuroscience methods. 2006;157(2):317–329.

[24] Sotero RC, Trujillo-Barreto NJ, Iturria-Medina Y, Carbonell F, Jimenez JC. Realistically coupled neural mass models can generate EEG rhythms. Neural computation. 2007;19(2):478–512.

[25] Moran RJ, Kiebel SJ, Stephan KE, Reilly R, Daunizeau J, Friston KJ. A neural mass model of spectral responses in electrophysiology. NeuroImage. 2007;37(3):706–720.

[26] Nunez PL. The brain wave equation: a model for the EEG. Mathematical Biosciences. 1974;21(3-4):279–297.

[27] Jirsa VK, Haken H. Field theory of electromagnetic brain activity. Physical review letters. 1996;77(5):960.

[28] Wright J, Liley D. Dynamics of the brain at global and microscopic scales: Neural networks and the EEG. Behavioral and Brain Sciences. 1996;19(2):285–295.

[29] Robinson PA, Rennie CJ, Wright JJ. Propagation and stability of waves of electrical activity in the cerebral cortex. Physical Review E. 1997;56(1):826.

[30] Robinson PA, Rennie C, Rowe DL, O'Connor S, Gordon, E. Multiscale brain modelling. Philosophical Transactions of the Royal Society B: Biological Sciences. 2005;360(1457):1043–1050.

[31] Abeysuriya RG, Rennie CJ, Robinson PA. Prediction and verification of nonlinear sleep spindle harmonic oscillations. Journal of Theoretical Biology. 2014;344:70–77.

[32] Breakspear M, Roberts JA, Terry JR, Rodrigues S, Mahant N, Robinson PA. A unifying explanation of primary generalized seizures through nonlinear brain modeling and bifurcation analysis. Cerebral Cortex. 2006;16(9):1296–1313.

[33] Zhao X, Robinson PA. Generalized seizures in a neural field model with bursting dynamics. Journal of computational neuroscience. 2015;39(2):197–216.

[34] Kerr CC, Rennie CJ, Robinson PA. Physiology-based modeling of cortical auditory evoked potentials. Biological cybernetics. 2008;98:171–184.

[35] Robinson P. Determination of effective brain connectivity from functional connectivity using propagator-based interferometry and neural field theory with application to the corticothalamic system. Physical Review E. 2014;90(4):042712.

[36] Robinson P, Rennie C, Rowe D. Dynamics of large-scale brain activity in normal arousal states and epileptic seizures. Physical Review E. 2002;65(4):041924.

[37] Griffiths JD, Bastiaens SP, Kaboodvand N. Whole-Brain Modelling: Past, Present, and Future. In: Computational Modelling of the Brain. Springer; 2022. p. 313–355.

[38] Saalmann YB, Pinsk MA, Wang L, Li X, Kastner S. The pulvinar regulates information transmission between cortical areas based on attention demands. science. 2012;337(6095):753–756.

[39] Lőrincz ML, Kékesi KA, Juhász G, Crunelli V, Hughes SW. Temporal framing of thalamic relay-mode firing by phasic inhibition during the alpha rhythm. Neuron. 2009;63(5):683–696.

[40] Hughes SW, Lőrincz ML, Blethyn K, Kékesi KA, Juhász G, Turmaine M, et al. Thalamic gap junctions control local neuronal synchrony and influence macroscopic oscillation amplitude during EEG alpha rhythms. Frontiers in psychology. 2011;2:193.

[41] Lopes da Silva F. Neural mechanisms underlying brain waves: from neural membranes to networks. Electroencephalography and clinical neurophysiology. 1991;79(2):81–93.

[42] Connors BW, Amitai Y. Making waves in the neocortex. Neuron. 1997;18(3):347–349.

[43] Bollimunta A, Chen Y, Schroeder CE, Ding M. Neuronal mechanisms of cortical alpha oscillations in awake-behaving macaques. Journal of Neuroscience. 2008;28(40):9976–9988.

[44] Nunez PL, Srinivasan R, et al. Electric fields of the brain: the neurophysics of EEG. Oxford University Press, USA; 2006.

[45] Lopes da Silva F. Dynamics of EEGs as signals of neuronal populations: models and theoretical considerations. Electroencephalography: Basic Principles, Clinical Applications and Related Fields, 4th edition. 1998; p. 76–92.

[46] Steriade M. Cellular substrates of brain rhythms. Electroencephalography: Basic principles, clinical applications, and related fields. 2005;5:31–83.

[47] Valdés-Hernández PA, Ojeda-González A, Martínez-Montes E, Lage-Castellanos A, Virués-Alba T, Valdés-Urrutia L, et al. White matter architecture rather than cortical surface area correlates with the EEG alpha rhythm. Neuroimage. 2010;49(3):2328–2339.

[48] Nunez PL, Cutillo BA. Neocortical dynamics and human EEG rhythms. Oxford University Press, USA; 1995.

[49] Nunez PL, Srinivasan R. A theoretical basis for standing and traveling brain waves measured with human EEG with implications for an integrated consciousness. Clinical neurophysiology. 2006;117(11):2424–2435.
