# Supplementary material for: A comprehensive investigation of intracortical and corticothalamic models of the alpha rhythm
Source: PLoS Comput Biol. 2025 Apr 10;21(4):e1012926. doi: 10.1371/journal.pcbi.1012926 (PMC12064047; doi:10.1371/journal.pcbi.1012926)
Supplement: Attachment — Submitted filename: Response_to_Reviewer.pdf [file pcbi.1012926.s011.pdf]

Dear Dr. Serre,

Thank you for your feedback on our manuscript. We appreciate the constructive comments from the editorial board regarding the length of the paper and its potential value to the community. In response to the board's suggestion, we have significantly shortened the manuscript, reducing its length by nearly half, while maintaining the integrity of our analysis. Below is a summary of the major revisions:

Background:

- Severely reduced the text by removing the explanation of other alpha hypotheses not explored in the paper, reducing the details on abnormal alpha oscillations and the discussion of conductance-based models.
- Rewrote the "Bridging scales" section for brevity.
- Moved the section “Tracing the roots of NPMs: early history” to the supplementary materials.

Methods:

- Moved the derivation of the stability of JR and LW models to the supplementary materials.
- For all model-related figures, we only retained the outputs of the numerical simulations, and moved the transfer function equations to the supplementary materials.

Results:

- Summarized the results of the "Topology", "Equations", and "Unified parameter table" sections into one concise paragraph within the “Comparative evaluation of the model” section.

Discussion:

- Removed the section "Alternative models of alpha rhythm beyond NPMs."
- Reworked and significantly shortened the "Summary of main findings."
- Severely reduced and rewrote the "Model limitations and critique" section.

We believe these changes have streamlined the manuscript while preserving its key insights. We hope the revised version meets the expectations of the editorial board and is suitable for review.

Thank you for considering our resubmission. We look forward to your feedback.

Sincerely,

Sorenza
