# Supplementary material for: A comprehensive investigation of intracortical and corticothalamic models of the alpha rhythm
Source: PLoS Comput Biol. 2025 Apr 10;21(4):e1012926. doi: 10.1371/journal.pcbi.1012926 (PMC12064047; doi:10.1371/journal.pcbi.1012926)
Supplement: Attachment — Submitted filename: Response_to_Reviewers.pdf [file pcbi.1012926.s012.pdf]

Response to Reviewer Comments

We would like to thank the Reviewers for the time and effort they invested in reviewing our manuscript *A comprehensive investigation of intracortical and corticothalamic models of the alpha rhythm*. We appreciate the constructive feedback and valuable suggestions provided, which have helped us improve the quality of our work.

Below, we provide detailed responses to each of the comments raised by the reviewers which are in italics:

Reviewer 1 comments and Responses:

1. In Section 3.2.1, the table comparing parameter values across the four models is a key strength of this study. However, the parameter values differ significantly across these models. A rational for parameter translation in the discussion section open more avenue for translatability of results across different models. As an example, a discussion subsection on how can model outputs be normalized for direct comparison could help direct future research in this field?

[AUTHORS]: We thank the reviewer's suggestion to enrich the discussion regarding parameter translatability across models. In this paper, we compared parameters by focusing on their effects—examining what occurs when their values increase or decrease—rather than their absolute magnitudes since they widely vary between the models. To further support future investigations, we have expanded this discussion with the following addition:

Added in discussion

Line 876: The challenges in associating model parameters with experimental observables described above also result in limitations regarding parameter translatability across models. In this work, we have therefore chosen to focus on comparing parameters of similar biological significance by analyzing their relative effects—specifically, how changes from their respective baseline values influence system behavior. While this approach provides valuable insights, the inability to make direct numerical comparisons across models, due to the significant differences in parameter scaling and baseline values, remains a major limitation. To enable such comparisons, future research could explore methods to normalize model outputs, for example by scaling impulse response functions and sigmoid curves so that their axes and ranges align across models. Alternatively, outputs such as oscillation amplitude or frequency could be standardized against experimental benchmarks or dimensionless parameter combinations. However, developing a systematic and biologically grounded normalization framework remains an open challenge that warrants further investigation.

2. The authors heavily rely on linear stability analysis and potential nonlinear behaviors (e.g., chaotic dynamics) and their implications are not explored. While linearization is a standard tool, the omission of nonlinear dynamics could overlook some critical dynamical phenomenon. This limitation should be discussed in the manuscript. Perhaps in the discussion section

[AUTHORS]: We appreciate the Reviewer's observation on this limitation and we agree it merits further details in the discussion section. We have added a section in the discussion to address this point:

Added in discussion

Line 912: To facilitate model comparison, our study primarily employed linear stability analysis to characterize model behavior near fixed points and to identify bifurcation types using phase planes (such as Hopf bifurcations). This approach enabled a systematic examination of the dynamical regimes within the model. However, we acknowledge that this method does not fully account for nonlinear dynamics, such as potential chaotic behavior, due to the approximations involved. For example, during linearization, the nonlinear sigmoid function is approximated by its slope at the operating point, effectively reducing it to a linear 'gain' parameter that reflects the population's sensitivity to small perturbations.This focus is appropriate for our study on alpha-band resting-state activity, which has been consistently shown to be well-described and valid under linear approximations in previous research as operating near critical bifurcations points (Stam et al., 1999; Rowe et al., 2004). In contrast, the study of nonlinear phenomena, such as seizure states or spike-wave activity, requires different analytical tools, as these dynamics depend heavily on nonlinearity (Zhao and Robinson, 2017). Future work could explore nonlinear behaviors more comprehensively by computing the Lyapunov exponent—a standard measure for quantifying chaos—or by simulating the model under diverse parameter settings to reveal complex temporal patterns (Spiegler et al., 2011; Dafilis et al., 2015; Roberts and Robinson, 2012b).

3. The authors mention that the "Mesoscopic activity is more challenging to measure directly". However, the LFP could be used as reasonable proxy for mesoscopic neural activity as it reflects the average synaptic activity of local neuronal population. Hence, a brief discussion of how LFPs could be used as a proxy for mesoscopic neural activity could provide more context.

[AUTHORS]: We appreciate the reviewer's comment regarding LFPs. While LFPs can serve as a proxy for measuring mesoscopic activity, they remain an invasive technique with limited spatial coverage. We agree that explicitly addressing this aspect in the introduction is important and have now included it accordingly.

Added in introduction

Line104: Although local field potentials (LFPs) provide a proxy for mesoscopic neural activity, they face limitations due to their invasiveness, limited spatial coverage, and the overlapping synaptic currents from diverse neural sources (Herreras, 2016). This complexity, combined with variability in recording and analysis methods, contributes to inconsistent interpretations of LFP-derived oscillatory rhythms.

Reviewer 2 comments and Responses:

Reviewer 2: The paper is an excellent compendium of four mathematical models used for studying alpha rhythms. The authors detail the comparative advantages/features of the

models in considerable detail. The study will no doubt be of value to specialists in the field. It is a pity that their analysis does not propose the experiments that would be necessary to select which of the models is the "correct" one, which weakens their work somewhat.

[AUTHORS]: Thank you for your thoughtful comment and suggestions. We acknowledge that a detailed explanation to researchers on whether and how to select a given model for their specific research application - including the question of how to disambiguate between competing theories of alpha - was not provided in our discussion. Ultimately the objective of the research agenda to which the present paper is our latest contribution, should indeed involve concrete and practical proposals of experimental tests for the different models. At the earliest stage of this project, it was our intention to develop some of these ideas. We did however find that this was more challenging than initially anticipated, primarily due to the inherent mathematical similarities between the competing frameworks, which result in overlapping predictions for resting-state dynamics. We would like to emphasize that in our manuscript, we provided Table 3, which is intended to address this in part by outlining the strengths and weaknesses of each model, allowing researchers to choose a model based on the specific goals of their study (e.g., ease of parameter manipulation, inclusion of thalamic structures, etc.). Additionally, we have added a section discussing potential experimental strategies for resolving this question in future work.

Added in discussion

Line 935: While this table provides guidance for selecting a model based on study goals, it is important to recognize that the mathematical similarity between models makes it difficult to unambiguously identify the "correct" one for representing resting-state alpha dynamics, as all can capture all or most features of empirical EEG resting-state dynamics across large domains of their parameter space. Future work could address this by applying controlled perturbations—such as pharmacological interventions, transcranial stimulation, or sensory inputs—to differentiate their evoked responses. That is, for each parameter configuration of all the models studied here, there is an associated evoked (impulse response) pattern that could be studied in conjunction with the resting (steady-state) behavior described in this paper. Expanding analyses to include cognitive or task-based measures or combining resting-state data with stimulated-state paradigms may also reveal unique, state-dependent features. These approaches offer promising pathways for resolving ambiguities and improving model evaluation.
